# Red vision in animals is broadly associated with lighting environment but not types of visual task

**DOI:** 10.1002/ece3.10899

**Published:** 2024-01-31

**Authors:** Bryony M. Margetts, Devi Stuart‐Fox, Amanda M. Franklin

**Affiliations:** ^1^ School of BioSciences The University of Melbourne Parkville Victoria Australia

**Keywords:** light environment, long wavelength sensitivity, red vision, visual ecology

## Abstract

Red sensitivity is the exception rather than the norm in most animal groups. Among species with red sensitivity, there is substantial variation in the peak wavelength sensitivity (λ_max_) of the long wavelength sensitive (LWS) photoreceptor. It is unclear whether this variation can be explained by visual tuning to the light environment or to visual tasks such as signalling or foraging. Here, we examine long wavelength sensitivity across a broad range of taxa showing diversity in LWS photoreceptor λ_max_: insects, crustaceans, arachnids, amphibians, reptiles, fish, sharks and rays. We collated a list of 161 species with physiological evidence for a photoreceptor sensitive to red wavelengths (i.e. λ_max_ ≥ 550 nm) and for each species documented abiotic and biotic factors that may be associated with peak sensitivity of the LWS photoreceptor. We found evidence supporting visual tuning to the light environment: terrestrial species had longer λ_max_ than aquatic species, and of these, species from turbid shallow waters had longer λ_max_ than those from clear or deep waters. Of the terrestrial species, diurnal species had longer λ_max_ than nocturnal species, but we did not detect any differences across terrestrial habitats (closed, intermediate or open). We found no association with proxies for visual tasks such as having red morphological features or utilising flowers or coral reefs. These results support the emerging consensus that, in general, visual systems are broadly adapted to the lighting environment and diverse visual tasks. Links between visual systems and specific visual tasks are commonly reported, but these likely vary among species and do not lead to general patterns across species.

## Graphical abstract

We conduct a comprehensive assessment of ecological drivers of red vision, across a broad range of taxa. Our results support the emerging consensus that, in general, visual systems are broadly adapted to the lighting environment and diverse visual tasks.

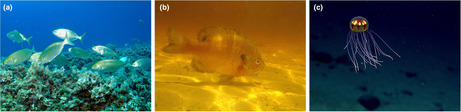

## 1 INTRODUCTION

Visual sensitivity to long wavelengths (>600 nm) or ‘red’ sensitivity is relatively rare across the animal kingdom, and we have little understanding of the factors favouring its evolution (but see Mollon, 1989; Murphy & Westerman, 2022b; Osorio & Vorobyev, 2005, 2008). Birds and reptiles commonly have a long wavelength sensitive (LWS) photoreceptor, but for other animal groups, including insects, fish and mammals, a LWS photoreceptor is uncommon (Kelber et al., 2003; Osorio & Vorobyev, 2005). Among the species with a LWS photoreceptor, there is substantial variation in the peak sensitivity of these photoreceptors (e.g. insects; van der Kooi et al., 2021). Often, the peak sensitivity (λ_max_) of the photoreceptor is below 600 nm, however sensitivity extends to wavelengths beyond the peak absorbance value. For example, the human red photoreceptor peaks at 562 nm and still has 30% relative absorbance at 635 nm (Bowmaker & Dartnall, 1980). Most animals have an LWS photoreceptor with λ_max_ below 600 nm, however, some insects, fish, reptiles and amphibians have LWS photoreceptors with λ_max_ beyond 600 nm (Escobar‐Camacho et al., 2020; Liebman & Entine, 1968; Martin et al., 2015; van der Kooi et al., 2021) including a butterfly with a LWS photoreceptor peaking at 660 nm (Ogawa et al., 2013). Among photoreceptor types, the LWS photoreceptor shows the greatest range of peak sensitivity across taxa; yet we have limited understanding of broad scale abiotic and biotic factors that may explain this variation.

Variation in red sensitivity among species can be produced via different mechanisms. Light detection is achieved via a visual pigment, which is an opsin protein coupled to a light‐sensitive retinal‐based chromophore. Changes to the opsin sequence or chromophore type can shift peak sensitivity. For example, using an A2 rather than A1 chromophore is one of the main mechanisms to shift LWS sensitivity to longer wavelengths in aquatic vertebrates (Carleton et al., 2008; Enright et al., 2015; Martin et al., 2015). In fact, many fish use both A1 and A2 chromophores and modulate the ratio in response to environmental conditions (reviewed in Corbo, 2021). Shifts to longer wavelengths via opsin or chromophore modification reduce chromophore activation energy and increases the susceptibility to activation by heat (Ala‐Laurila et al., 2004; Barlow, 1957; Luo et al., 2011). This reduces the signal to noise ratio, which can be particularly problematic in low light conditions when the signal is low, and is thought to restrict the upper limit of visual pigment absorption (Cronin et al., 2014). Another mechanism to shift spectral sensitivity to wavelengths longer than the peak absorbance of the opsin is the use of filtering or screening pigments. This mechanism is commonly observed in insects to produce photoreceptors with peak sensitivity greater than 600 nm (Ogawa et al., 2013; Satoh et al., 2017; Wakakuwa et al., 2004). By narrowing the spectral sensitivity of the photoreceptor, filtering pigments decrease the absolute sensitivity. In this study, we are interested in sensitivity to red wavelengths (i.e. ≥600 nm) regardless of the mechanism. Thus, we use ‘LWS’ to refer to a photoreceptor with λ_max_ ≥ 550 nm, because this would provide sensitivity to red wavelengths.

**FIGURE 1:**
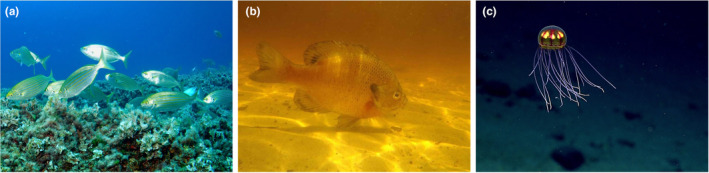
An illustrative example of the spectral properties of light in different aquatic environments. (a) A blue light dominated environment, such as shallow, clear reefs (Image: Lars Behnke). (b) A red light dominated environment, such as turbid waters (Image: Tom Tetzner, U.S. Fish and Wildlife Service). (c) A low illumination, deep water environment (note that the animal is illuminated by ROV lights; Image: NOAA Office of Ocean Exploration).

The primary hypothesis to explain variation in photoreceptor sensitivities is that they are tuned to the light environment. In aquatic environments, the selective transmission of blue light in deep, clear oceanic waters corresponds to blue‐shifted visual sensitivity in fish (Denton & Warren, 1956; Douglas & Partridge, 2011; Schweikert et al., 2019) as well as other organisms living in deeper waters (e.g. crustaceans; Frank et al., 2012; Marshall, Cronin, & Frank, 2003). Water strongly absorbs red wavelengths and red light is primarily only present in shallow waters (<10 m; Bowling et al., 1986; Marshall, Jennings, et al., 2003; Warrant & Johnsen, 2013). In turbid waters, the suspended particles increase the proportion of red light by more strongly attenuating shorter wavelengths than longer wavelengths of light (Loew & McFarland, 1990; Lythgoe, 1972) (Figure 1). This is associated with red‐shifted vision in many fish (Carleton et al., 2005; Corbo, 2021). Within terrestrial environments, small gaps in canopy cover result in higher amounts of red shifted light than open or closed environments because these gaps contain lower proportions of blue light scattered by the atmosphere and higher proportions of direct sunlight (Endler, 1993). A moonless night is around 100 million times darker than a bright sunny day, dramatically reducing the signal to noise ratio in the visual pathway (Kelber & Roth, 2006; Osorio & Vorobyev, 2005; Warrant & Johnsen, 2013). Therefore, thermal noise will be more important in dim light (Osorio & Vorobyev, 2005) and will have a greater impact on longer wavelength photoreceptors because as λ_max_ increases the energy barrier for thermal isomerisation falls (Ala‐Laurila et al., 2004; Luo et al., 2011). Based on characteristics of the light environment, we might therefore expect longer wavelength sensitivity in terrestrial than aquatic environments, in turbid than clear waters, in intermediate than open or closed canopy environments and in diurnal than nocturnal species. Evidence for predicted relationships between visual sensitivities and light environment is mixed (Briscoe & Chittka, 2001; Fleishman et al., 1997; Lind et al., 2017; Loew & McFarland, 1990; Partridge, 1989). However, a comprehensive recent study found that terrestrial species have longer wavelength sensitivity than aquatic species and of the terrestrial species, those occupying closed canopy habitats have longer wavelength sensitivity than those from open habitats (if generalist species are excluded; Murphy & Westerman, 2022b). This study examined photoreceptors with the shortest and longest wavelength sensitivity in each species, irrespective of photoreceptor type, so the longest λ_max_ for the majority of species corresponds to a photoreceptor with little sensitivity to red wavelengths. Whether general relationships exist between red sensitivity (LWS peak sensitivity) and habitat light remains an open question.

A second hypothesis to explain variation in visual sensitivities is that they are tuned to certain types of visual task, such as foraging or signalling. Numerous animals use red sexual signals during mate choice (Amundsen & Forsgren, 2001; Belliure et al., 2018; Hill, 2006; Kwiatkowski & Sullivan, 2002) and shifts in long wavelength sensitivity may improve discrimination of these signals (Carleton et al., 2005; Stieb et al., 2023). Within some animal groups, long wavelength sensitivity has been shown to improve discrimination of objects, such as fruit, flowers or conspecifics, from the background (Mollon, 1989; Stieb et al., 2023; Sumner & Mollon, 2000; Wang et al., 2022), and for *Papilio aegeus* butterflies, a LWS photoreceptor can also improve identification of young leaves suitable for oviposition from old, unsuitable leaves (Kelber, 1999). Both Kelber (1999) and Wang et al.'s (2022) studies find that a red photoreceptor can help to distinguish colours other than red if there is variation in reflectance at long wavelengths. In addition, colour vision models suggest that shifting LWS peak sensitivity to longer wavelengths can improve discrimination of resources or mates (Stieb et al., 2023; Wang et al., 2022), but improvements may be small (Lind et al., 2017). Most examples linking LWS photoreceptor peak sensitivity with visual task concern specific species and it remains unclear whether general patterns exist between broad categories of visual tasks, such as foraging or signalling, and LWS photoreceptor sensitivity.

We investigated visual tuning the of the LWS photoreceptor across a wide range of taxa that show variation in the presence and peak sensitivity of the LWS photoreceptor. This included insects, crustaceans, arachnids, amphibians, reptiles, fish, sharks and rays. Birds and mammals were excluded a priori because these groups show limited variation in LWS sensitivity. We collated a list of species with physiological evidence for a LWS photoreceptor sensitive to red wavelengths (i.e. λ_max_ ≥ 550 nm). This list substantially expands the species with λ_max_ ≥ 550 nm independently compiled by Murphy and Westerman (2022b). For each species, we recorded abiotic and biotic factors that may be associated with increased peak sensitivity of the LWS photoreceptor. Specifically, we tested four predictions based on visual tuning to the light environment: (1) terrestrial species will have higher λ_max_ than aquatic species; (2) within aquatic species those living in turbid, shallow waters will have higher λ_max_; (3) within terrestrial species, those living in habitats with intermediate levels of canopy cover will have greater λ_max_ than those associated with closed or open habitats and (4) diurnal terrestrial animals will have greater λ_max_ than nocturnal species. To explore whether visual systems may be tuned to general visual tasks, we examined whether λ_max_ is associated with proxies for signalling and foraging tasks. Specifically, we tested whether λ_max_ is higher for species that have red morphological features, or are sexual dichromatic (proxies for interspecific and/or intraspecific signalling) or are associated with flowers or coral reefs (related to foraging ecology). Together these results provide insight into the evolution and function of red sensitivity.

## 2 METHODS

### 2.1 Literature search

We focused on animal groups with known variation in the presence and peak sensitivity of the long wavelength photoreceptor: insects, crustaceans, arachnids, amphibians, reptiles, fish, sharks and rays. Birds and mammalian species were excluded a priori because these groups show limited variation in long wavelength sensitivity; birds commonly have a long wavelength photoreceptor but peak sensitivity is similar among species (λ_max_ approximately 560 to 570 nm; Hart, 2001; Hart & Hunt, 2007) and mammals generally lack long wavelength photoreceptors, although there are some exceptions (e.g. some primates; Jacobs, 2009; Osorio & Vorobyev, 2005). For this study, we defined a LWS photoreceptor as one with a peak sensitivity (λ_max_) ≥ 550 nm because this provides sensitivity to red wavelengths (>600 nm).

To document species with LWS photoreceptors, we first identified review articles that provide details of spectral sensitivity for the groups of interest (i.e. Aksoy & Camlitepe, 2018; Briscoe & Chittka, 2001; Carleton, 2009; Hart et al., 2020; Jokela‐Määttä et al., 2007; Kelber et al., 2003; Martin et al., 2015; van der Kooi et al., 2021). To supplement these review articles, we also conducted a Web of Science search. The search terms we used were “long wavelength spectral sensitivity”, “(spectral sensitivity) AND (MSP OR microspectrophotometry)”, “(spectral sensitivity) AND (ERG OR electroretinogram)” and “(spectral sensitivity) AND intracellular”. We filtered the search to only those under the zoology and ecology categories. After removing duplicates, additional exclusion criteria were applied. We included only studies where λ_max_ was recorded through electroretinogram (ERG), microspectrophotometry (MSP), intracellular recording or partial bleaching. If multiple studies were identified for a species and the λ_max_ differed between these studies, the most current and/or rigorous was recorded (i.e. preferentially intracellular recording, followed by ERG and MSP, or those with larger sample sizes). This search was completed in January 2022 and produced 34 articles identifying an additional 79 species with a LWS photoreceptor.

### 2.2 Data extraction and processing

For each species identified we recorded: λ_max_ of all photoreceptors; the method used to measure λ_max_; species name (including population or sub‐group information where applicable) and higher classification information; life stage (adult/immature) and sex. Several data points required some additional processing to determine the λ_max_ of the LWS photoreceptor. First, where λ_max_ was given as a range we recorded the mean of the range. Second, we identified four fish species with different λ_max_ among populations found in different habitats. To account for these duplications, a random effect of species was included in the statistical models. Third, recordings that were from juvenile or immature life stages were removed (*n* = 18) and duplications due to recordings from both males and females were removed (LWS λ_max_ was the same for both sexes). Finally, in one fish, λ_max_ values were reported based on the A1/A2 chromophore ratios, which correspond to different visual sensitivities (Escobar‐Camacho et al., 2019). Many fish use a mix of A1 and A2 chromophores and can change this ratio in response to environmental conditions (Carleton et al., 2008; Enright et al., 2015). Therefore, for this record we calculated a single λ_max_ by multiplying the ratio of each pigment (A1/A2) with its corresponding λ_max_ and summing these values.

To obtain information about habitat, morphology and behaviour, we searched the primary literature and online databases. For all species, we recorded broad scale habitat (terrestrial, aquatic or semi‐aquatic) and sub‐habitat (terrestrial: open, intermediate or closed; aquatic: shallow‐turbid, shallow‐clear or deep). For terrestrial species, open habitat consisted of grasslands, scrublands, canopy dwelling species or habitats with few to no trees, closed habitats were dense forests such as rainforests and intermediate habitats included generalist species or open forests. For aquatic species, those regularly found at depths >500 m were classified as deep whereas those closer to the surface (<500 m) were shallow. Water turbidity was based on recent descriptions of water clarity for species found in specific water bodies. For species with a larger range, turbid water was assigned to animals inhabiting rivers, lakes, ponds and estuaries, and clear water was assigned to open marine environments. We also documented activity period (diurnal or nocturnal) for terrestrial species. To examine spectral tuning to visual tasks, we recorded morphological characteristics including the presence of red colouration and sexual dimorphism in hue and/or colour intensity (human visible colours) using primary literature and photographs. For terrestrial species, we documented flower association (present or absent), based upon whether a species was a pollinator, florivore or if it was documented as a flower visiting species. For aquatic species we documented coral or reef association (present or absent), based upon information reported by FishBase and photo documentation. Information obtained through primary literature or trusted databases (i.e. Animal Diversity Web (ADW), World Register of Marine Species (WoRMs) and FishBase) was prioritised over photographic information. If relevant information could not be found, that data point was excluded from relevant analyses.

### 2.3 Statistical analysis

We used linear mixed models (LMM) to determine the relationship between the λ_max_ of the long wavelength photoreceptor and habitat, morphology and behaviour. In such analyses, it is important to account for phylogenetic relationships to prevent pseudo‐replication. Given the extremely broad and patchy phylogenetic distribution of taxa in this study, branch length information for a derived phylogeny (e.g. from Open Tree of Life, tree.opentreeoflife.org) is inaccurate. In our study, phylogenetic pseudo‐replication is largely due to multiple closely related species within families, whereas most of the families are distantly related. Thus, phylogenetic non‐independence can be accounted for by including ‘family’ as a random effect. This accounts for non‐independence of species within the same family and assumes that evolutionary origins of wavelength sensitivity are largely independent among families, which is reasonable given the phylogenetic sampling within our dataset. For all models, the peak absorbance of the LWS photoreceptor (λ_max_) was the dependant variable and for models involving aquatic organisms ‘species’ was also included as a random effect to account for multiple populations with different λ_max_. All analyses were conducted in R version 4.1.2 (R Core Team, 2022) using the lme4 package (Bates et al., 2015) and significance of each predictor variable was assessed using marginal hypothesis tests, implemented using the ANOVA command from the car package (Fox & Weisberg, 2019).

We ran four LMMs on different subsets of the dataset. The first model was conducted using the full dataset and included broad‐scale habitat (terrestrial/aquatic/semi‐aquatic), sexual dimorphism in body colouration hue (present/absent), sexual dimorphism in body colouration intensity (present/absent) and presence of a red morphological feature (present/absent). The second model included only aquatic species and the fixed effects were sub‐habitat type (shallow turbid/shallow clear/deep) and coral reef association (present/absent). The third model included only terrestrial species and the fixed effects were terrestrial sub‐habitat (open/intermediate/closed) and activity time (diurnal/nocturnal). We also included an interaction between sub‐habitat and activity time because sub‐habitat may only influence peak sensitivity for diurnal animals. Only insects exhibited substantial variation in flower association, thus, the fourth model investigated flower association in insects and included flower association (present/absent) as a fixed effect. For some categories, we identified fewer than 10 records of species with a LWS photoreceptor. We ran these models with and without those groups to assess consistency of results. Specifically, model 1 (all data) was run with and without semi‐aquatic species (*n* = 7), model 2 (aquatic species) was run with and without deep sea species (*n* = 8) and model 3 (terrestrial species) was run with and without species from closed habitats (*n* = 7). For all analyses, results were qualitatively similar, so we report results of the full models.

## 3 RESULTS

In total, we identified 89 peer‐reviewed journal articles documenting spectral sensitivities from nine classes including Actinopterygii, Amphibia, Arachnida, Branchiopoda, Chondrichthyes, Hyperoartia, Insecta, Malacostraca and Reptilia (Table S1). From these articles we extracted 174 records of LWS photoreceptors (λ_max_ ≥ 550 nm). There were 164 different species, including 52 insects, 49 fish, 30 reptiles, 17 sharks and rays, eight crustaceans, six amphibians and two arachnids.

### 3.1 Spectral tuning to the light environment

**FIGURE 2:**
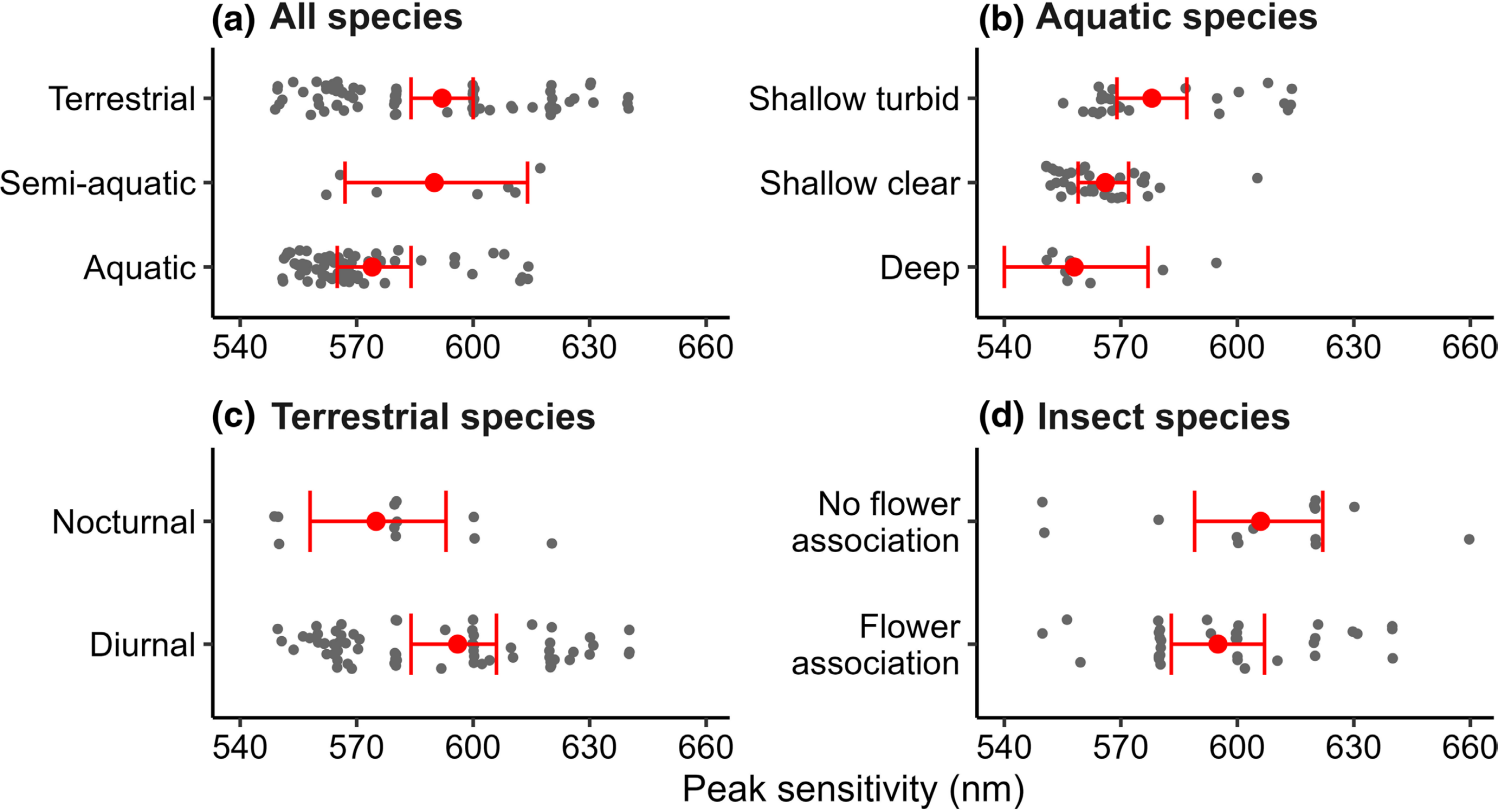
Effect of lighting environment and visual tasks on peak sensitivity (λ_max_) of the long wavelength sensitive (LWS) photoreceptor. (a) terrestrial species have longer λ_max_ than aquatic species. (b) Species from shallow, turbid water have longer λ_max_ than species from shallow clear water or deep water. (c) Diurnally active species have longer λ_max_ than nocturnally active species. (d) Flower association did not influence λ_max_ of the LWS photoreceptor. Small grey points represent λ_max_ from each species, and large red points represent estimated marginal means ± 95% confidence intervals.

Of the species included in the analysis, there were 78 terrestrial, 67 aquatic and seven semi‐aquatic records of species or populations with a LWS photoreceptor. We found that terrestrial species had a mean peak sensitivity of 592 nm (95% CI = 584, 600 nm), approximately 18 nm higher than aquatic species (574 nm, 95% CI = 565, 584 nm; χ^2^ = 9.49, *p* = .009; Figure 2; Table 1). Semi‐aquatic species did not differ significantly from either group (Figure 2).

**TABLE 1:** Statistical results for each model testing the association between predictor variables and peak sensitivity of the long wavelength photoreceptor.

Model/data	Predictor	χ^2^	df	*p*‐value
(1) All data	Habitat	9.49	1	**.009**
	Sexual dimorphism – hue	0.12	1	.729
	Sexual dimorphism – intensity	1.51	1	.220
	Red feature	2.25	1	.134
(2) Aquatic only	Sub‐habitat (Aquatic)	10.28	2	**.006**
	Reef/coral association	2.66	1	.103
(3) Terrestrial only	Sub‐habitat (Terrestrial)	0.89	3	.642
	Activity	4.83	1	**.028**
	Activity × Sub‐habitat interaction	2.98	2	.225
(4) Insects only	Flower association	1.28	1	.258

*Note*: Marginal hypothesis tests were conducted to test the significance of each predictor in each model. *p*‐Values in bold indicate values <.05.

Of the aquatic species with a LWS photoreceptor, we found 35 from shallow clear waters, 24 from shallow turbid waters and eight from deep waters. Aquatic species living in turbid environments had the highest mean λ_max_ at 578 nm (95% CI = 569, 587 nm), 12 nm longer those living in clear water (566 nm, 95% CI = 559, 572 nm) and 16 nm longer than those living in deep water (558 nm, 95% CI = 540, 577 nm; χ^2^ = 10.28, *p* = .006; Figure 2, Table 1).

Within terrestrial species, 67 species were diurnal compared to just 11 nocturnal species, of which the majority were moths or beetles. Diurnal species had a mean peak sensitivity of 595 nm (95% CI = 584, 606 nm), 20 nm longer than the nocturnal group with peak sensitivity averaging 575 nm (95% CI = 558, 593 nm; χ^2^ = 4.83, *p* = .028; Figure 2 Table 1). Most terrestrial animals with a long wavelength photoreceptor were from open (*n* = 31) or intermediate habitats (*n* = 41), with only seven from closed habitats. There was no difference in the LWS photoreceptor peak sensitivity among species from these different habitats (Table 1).

### 3.2 Spectral tuning to visual tasks

Of the species included in the analysis, roughly half of the species or populations possessed red colouration (72 with red colouration and 80 without red colouration). Of the 145 records where colouration information for each sex was available, eight species were sexually dimorphic only in hue, 12 species were sexually dimorphic only in colour intensity, and 43 species were sexually dimorphic in both hue and intensity. We detected no difference in peak sensitivity of the long wavelength photoreceptor related to the presence of red colouration or sexual dimorphism in hue or intensity (Table 1).

Of the 67 aquatic species, 13 were associated with coral reefs, and this association did not correlate with peak sensitivity of the LWS photoreceptor (Table 1).

Of the 49 insect species with a LWS photoreceptor, 36 were associated with flowers and this association did not correlate with LWS λ_max_ (Figure 2; Table 1). The 13 insect species that were not associated with flowers tended to be predatory insects or did not feed at all during their adult life stage.

## 4 DISCUSSION

Red sensitivity is relatively uncommon among animals. Of those with a LWS photoreceptor there is substantial variation in its peak wavelength sensitivity (λ_max_), raising the question, why? We identified 164 species with a LWS photoreceptor (λ_max_ ≥ 550 nm) across a range of taxa and found that several variables describing light environment were associated with peak sensitivity of the LWS photoreceptor. Specifically, terrestrial species had higher LWS λ_max_ than aquatic species, diurnal terrestrial species had higher LWS λ_max_ than nocturnal terrestrial species and aquatic species in turbid shallow habitats had higher LWS λ_max_ than those in clear or deep waters. Contrary to expectations (Endler, 1993; Murphy & Westerman, 2022b), peak sensitivity of the LWS photoreceptor was not higher for terrestrial species in intermediate habitats compared to open or closed. We also found no evidence supporting visual tuning to visual tasks broadly related to signalling and foraging. These patterns align with an emerging consensus that visual systems are broadly tuned to the light environment and to perform diverse visual tasks. Visual systems may be tuned to perform specific visual tasks related to ecology or behaviour, but these are likely to be idiosyncratic (Lind et al., 2017; Osorio & Vorobyev, 2008), obscuring general patterns across species.

### 4.1 Spectral tuning to the light environment

Variation in peak sensitivity of the LWS photoreceptor was associated with habitat, likely due to differences in the spectral composition of illumination across habitats. In most terrestrial environments long wavelengths of light are prevalent (Endler, 1993; Warrant & Johnsen, 2013), whereas in aquatic environments long wavelengths from sunlight are rapidly attenuated and very little red light remains beyond 10 m depth (Bowling et al., 1986; Marshall, Jennings, et al., 2003; Warrant & Johnsen, 2013). Our results reflect this difference in the presence of long wavelengths, with aquatic species having lower λ_max_ of the LWS photoreceptor on average than terrestrial species. For aquatic species, the longest peak sensitivity was 614 nm, suggesting that further shifts in LWS sensitivity to longer wavelengths provides minimal improvements in discrimination or sensitivity. Several terrestrial species possessed LWS photoreceptors with peak sensitivity >615 nm, up to 660 nm. These LWS photoreceptors may function to improve discrimination of resources (e.g. oviposition sites, conspecifics, food; Kelber, 1999; Wang et al., 2022). The upper limit for terrestrial species may be due to limited improvements in discrimination of natural colours (Wang et al., 2022) and poor signal to noise ratio due a to increasing susceptibility of the photoreceptor to be activated by thermal noise (Koskelainen et al., 2000; Luo et al., 2011).

Within aquatic environments, peak sensitivity of the LWS receptor was greater for species from turbid water compared to those from shallow, clear water or deep water. Turbid waters tend to have a higher proportion of long wavelength light compared to clear water because the suspended particles attenuate shorter wavelengths (Jones et al., 2021; Sundarabalan et al., 2016). Numerous studies have documented that species inhabiting turbid waters tend to have red‐shifted photoreceptors compared to species in clear waters (Carleton, 2009; Carleton et al., 2020; Corbo, 2021; Lythgoe et al., 1994; Nagloo et al., 2016) and many of these species achieve this by changing the chromophore used or the ratio of A1/A2 chromophores (Corbo, 2021). Our findings indicate that similar patterns occur across a broad range of aquatic species, including fish, crustaceans, sharks and rays. We also identified eight deep water species with a LWS photoreceptor, despite little downwelling light penetrating beyond 500 m deep (Lythgoe, 1988; Warrant & Johnsen, 2013). Three of these were mysid crustaceans, which often vertically migrate to shallow waters to feed at night. The LWS photoreceptor may improve discrimination in shallow waters or assist in habitat choice, but this remains to be tested. The remaining five species were stomiid dragon fish that all have red bioluminescence (Bowmaker et al., 1988; Crescitelli, 1989; O'Day & Fernandez, 1974; Partridge & Douglas, 1995). Red bioluminescence is relatively uncommon and likely provides them with a ‘secret signalling system’ that potential predators cannot see (Douglas et al., 1999). Various deep‐water species have visual systems specialised to detect bioluminescence (Frank et al., 2012, 2016; Turner et al., 2009; Warrant & Locket, 2004), providing an example of visual capabilities tuned to visual tasks such as intraspecific signalling or prey detection.

Unlike the trends across aquatic habitats, we detected no difference in peak sensitivity of the LWS photoreceptor across terrestrial habitats. This result differs from predictions that long wavelength sensitivity may be beneficial in forests with intermediate canopy cover because illumination is red shifted compared to open habitats (Endler, 1993). A recent review supported this hypothesis, finding that specialist species from closed or intermediate habitats have sensitivity to longer wavelengths of light than specialist species from open habitats (Murphy & Westerman, 2022b). Our results may differ due to the species included in our analysis and our focus on only the LWS photoreceptor, rather than maximum sensitivity of any photoreceptor. We identified 82 terrestrial species with an LWS photoreceptor, excluding birds and mammals, whereas Murphy and Westerman identified 27 terrestrial species with a LWS photoreceptor (λ_max_ ≥ 550 nm), including 13 birds and four mammals. The terrestrial species in our dataset were predominantly insects and reptiles and the results align with previous studies finding no correlation between spectral sensitivity and the terrestrial photic environment in these groups (Briscoe & Chittka, 2001; Fleishman et al., 1997). Furthermore, lighting environment may influence the presence of a LWS photoreceptor, rather than the peak sensitivity. Both datasets support this hypothesis; we identified more species with an LWS photoreceptor from intermediate or closed habitats (*n* = 47) compared to open habitats (*n* = 31), and Murphy and Westerman (2022a) indicates 57% of species from closed or intermediate habitats have an LWS photoreceptor (λ_max_ ≥ 550 nm) compared to only 27% of species from open habitats. Taken together, these results suggest that a LWS photoreceptor may be beneficial in intermediate or closed terrestrial habitats but there is limited evidence for consistent correlations between LWS peak sensitivity and habitat for terrestrial animals.

We identified relatively few nocturnal terrestrial species with a LWS receptor (*n* = 11), however these species tended to have shorter LWS λ_max_ than diurnal species. This shift to shorter wavelengths may improve the signal to noise ratio in low light conditions. The impact of thermal noise is greater for photoreceptors with higher λ_max_, due to the activation energy threshold decreasing with increasing λ_max_ (Ala‐Laurila et al., 2004; Barlow, 1957; Luo et al., 2011). Several studies have documented that nocturnal species have LWS photoreceptors with λ_max_ shifted to shorter wavelengths compared to diurnal species (Eguchi et al., 1982; Ellingson et al., 1995; Hart & Vorobyev, 2005; Potier et al., 2020), and our results support these findings.

### 4.2 Spectral tuning to visual tasks

Parameters related to the colour of resources or conspecifics were not associated with the peak sensitivity of the LWS photoreceptor. Species associated with flowers or coral reefs, those with red colouration or those with sexual dimorphism in body colour or intensity did not possess sensitivity to longer wavelengths compared to species without these characteristics. This result is consistent with previous work suggesting that long wavelength photoreceptors have not evolved in response to signals but instead may have evolved in response to common colours within the background (Osorio & Vorobyev, 2005; Stieb et al., 2023; Sumner & Mollon, 2000; Surridge et al., 2003). For example, the LWS photoreceptor of terrestrial animals may be tuned to the reflectance of foliage (Lythgoe, 1979; Osorio & Vorobyev, 2005). In this case, a long wavelength photoreceptor could function to detect variation among leaves (e.g. species of plant, young vs. old leaves; Kelber, 1999; Lythgoe, 1979), identify resources against a foliage background (e.g. flowers, fruit, conspecifics; Sumner & Mollon, 2000; Wang et al., 2022) and detect differences between leaves and other natural objects (e.g. bark, soil, dead vegetation; Osorio & Bossomaier, 1992; Osorio & Vorobyev, 2005). Furthermore, due to the diverse functions a visual system must perform beyond finding resources or conspecifics, it is perhaps unlikely to find associations between peak sensitivity and one visual task (Lind et al., 2017).

Despite finding no relationship between peak sensitivity and ecological variables, there are several species within our dataset that exhibit behaviours associated with long wavelength vision. Many animals show female preference for red colouration during mate choice, including the red dewlap colouration in *Anolis carolinensis* lizards (Sigmund, 1983) or the orange‐red fin and body colouration in mollies (*Poecilia* spp; Endler, 1984; Houde, 1987). Similarly, in many species males display red colouration around the breeding season or at sexual maturation (e.g. Bakker & Mundwiler, 1994; Meinertzhagen et al., 1983; Vranken et al., 2020). Long wavelength sensitivity in butterflies may also improve discrimination of courtship signals (Ogawa et al., 2013) and has been shown to improve discrimination of young and old leaves for selection of oviposition sites (Kelber, 1999). In beetles, long wavelength sensitivity is relatively rare, but is present in some families (van der Kooi et al., 2021) and is more common in species with flower associations (Sharkey et al., 2021). These examples highlight that long wavelength sensitivity is important for specific tasks, even if tuning of peak sensitivity is not correlated with ecological variables more broadly. For many species, we still have limited understanding of how they process visual information, whether the LWS photoreceptor contributes to colour vision or how vision guides behaviours. Further investigations into colour vision, behaviour and ecology of species with a LWS photoreceptor will likely provide new insights into the function of red sensitivity.

## AUTHOR CONTRIBUTIONS

**Bryony M. Margetts:** Conceptualization (equal); data curation (equal); formal analysis (lead); investigation (lead); methodology (equal); software (equal); validation (supporting); visualization (equal); writing – original draft (lead); writing – review and editing (equal). **Devi Stuart‐Fox:** Conceptualization (equal); formal analysis (supporting); investigation (supporting); methodology (equal); project administration (supporting); software (equal); supervision (supporting); writing – original draft (supporting); writing – review and editing (equal). **Amanda M. Franklin:** Conceptualization (lead); data curation (equal); formal analysis (supporting); investigation (supporting); methodology (equal); project administration (lead); software (equal); supervision (lead); validation (lead); visualization (equal); writing – original draft (supporting); writing – review and editing (equal).

## FUNDING INFORMATION

AMF is supported by a Melbourne Postdoctoral Fellowship awarded by The University of Melbourne. DSF was funded by the Australian Research Council (FT180100216; DP190102203).

## CONFLICT OF INTEREST STATEMENT

We declare we have no competing interests.

### OPEN RESEARCH BADGES

This article has earned an Open Data badge for making publicly available the digitally‐shareable data necessary to reproduce the reported results. The data is available at https://doi.org/10.5061/dryad.8gtht76ts.

## Supporting information


Table S1:

## Data Availability

Data and analysis code are available on Dryad: https://datadryad.org/stash/share/WpeH1ZRBmIosfznVo10MwkoAqnD9NWzKeR4hsPkQ3R4 (link for peer review); https://doi.org/10.5061/dryad.8gtht76ts.

## References

[ece310899-bib-0001] Aksoy, V. , & Camlitepe, Y. (2018). Spectral sensitivities of ants–a review. Animal Biology, 68, 55–73.

[ece310899-bib-0002] Ala‐Laurila, P. , Pahlberg, J. , Koskelainen, A. , & Donner, K. (2004). On the relation between the photoactivation energy and the absorbance spectrum of visual pigments. Vision Research, 44, 2153–2158. 15183682 10.1016/j.visres.2004.03.031

[ece310899-bib-0003] Amundsen, T. , & Forsgren, E. (2001). Male mate choice selects for female coloration in a fish. Proceedings of the National Academy of Sciences of the United States of America, 98, 13155–13160. 11606720 10.1073/pnas.211439298 PMC60840

[ece310899-bib-0004] Bakker, T. C. , & Mundwiler, B. (1994). Female mate choice and male red coloration in a natural three‐spined stickleback (*Gasterosteus aculeatus*) population. Behavioral Ecology, 5, 74–80.

[ece310899-bib-0005] Barlow, H. B. (1957). Purkinje shift and retinal noise. Nature, 179, 255–256. 10.1038/179255b0 13407693

[ece310899-bib-0006] Bates, D. , Maechler, M. , Bolker, B. , & Walker, S. (2015). Fitting linear mixed‐effects models using lme4. Journal of Statistical Software, 67, 1–48. 10.18637/jss.v067.i01

[ece310899-bib-0007] Belliure, J. , Fresnillo, B. , & Cuervo, J. J. (2018). Male mate choice based on female coloration in a lizard: The role of a juvenile trait. Behavioral Ecology, 29, 543–552.

[ece310899-bib-0008] Bowling, L. C. , Steane, M. S. , & Tyler, P. A. (1986). The spectral distribution and attenuation of underwater irradiance in Tasmanian inland waters. Freshwater Biology, 16, 313–335. 10.1111/j.1365-2427.1986.tb00974.x

[ece310899-bib-0009] Bowmaker, J. , Dartnall, H. , & Herring, P. (1988). Longwave‐sensitive visual pigments in some deep‐sea fishes: Segregation of ‘paired’ rhodopsins and porphyropsins. Journal of Comparative Physiology A, 163, 685–698.

[ece310899-bib-0010] Bowmaker, J. K. , & Dartnall, H. J. (1980). Visual pigments of rods and cones in a human retina. The Journal of Physiology, 298, 501–511. 10.1113/jphysiol.1980.sp013097 7359434 PMC1279132

[ece310899-bib-0011] Briscoe, A. D. , & Chittka, L. (2001). The evolution of color vision in insects. Annual Review of Entomology, 46, 471–510. 10.1146/annurev.ento.46.1.471 11112177

[ece310899-bib-0012] Carleton, K. (2009). Cichlid fish visual systems: Mechanisms of spectral tuning. Integrative Zoology, 4, 75–86. 21392278 10.1111/j.1749-4877.2008.00137.x

[ece310899-bib-0013] Carleton, K. L. , Escobar‐Camacho, D. , Stieb, S. M. , Cortesi, F. , & Marshall, N. J. (2020). Seeing the rainbow: Mechanisms underlying spectral sensitivity in teleost fishes. Journal of Experimental Biology, 223, jeb193334. 32327561 10.1242/jeb.193334 PMC7188444

[ece310899-bib-0014] Carleton, K. L. , Parry, J. W. L. , Bowmaker, J. K. , Hunt, D. M. , & Seehausen, O. L. E. (2005). Colour vision and speciation in Lake Victoria cichlids of the genus *Pundamilia* . Molecular Ecology, 14, 4341–4353. 10.1111/j.1365-294X.2005.02735.x 16313597

[ece310899-bib-0015] Carleton, K. L. , Spady, T. C. , Streelman, J. T. , Kidd, M. R. , McFarland, W. N. , & Loew, E. R. (2008). Visual sensitivities tuned by heterochronic shifts in opsin gene expression. BMC Biology, 6, 1–14. 18500997 10.1186/1741-7007-6-22 PMC2430543

[ece310899-bib-0016] Corbo, J. C. (2021). Vitamin A1/A2 chromophore exchange: Its role in spectral tuning and visual plasticity. Developmental Biology, 475, 145–155. 10.1016/j.ydbio.2021.03.002 33684435 PMC8900494

[ece310899-bib-0017] Crescitelli, F. (1989). The visual pigments of a deep‐water malacosteid fish. Journal of the Marine Biological Association of the United Kingdom, 69, 43–51.

[ece310899-bib-0018] Cronin, T. W. , Johnsen, S. , Marshall, N. J. , & Warrant, E. J. (Eds.). (2014). Visual pigments and photoreceptors. In Visual ecology (pp. 37–65). Princeton University Press.

[ece310899-bib-0019] Denton, E. , & Warren, F. (1956). Visual pigments of deep‐sea fish. Nature, 178, 1059. 10.1038/1781059a0 13378532

[ece310899-bib-0020] Douglas, R. , Partridge, J. C. , Dulai, K. S. , Hunt, D. M. , Mullineaux, C. W. , & Hynninen, P. H. (1999). Enhanced retinal longwave sensitivity using a chlorophyll‐derived photosensitiser in Malacosteus Niger, a deep‐sea dragon fish with far red bioluminescence. Vision Research, 39, 2817–2832. 10492812 10.1016/s0042-6989(98)00332-0

[ece310899-bib-0021] Douglas, R. H. , & Partridge, J. C. (2011). VISION | visual adaptations to the Deep Sea. In A. P. Farrell (Ed.), Encyclopedia of fish physiology (pp. 166–182). Academic Press.

[ece310899-bib-0022] Eguchi, E. , Watanabe, K. , Hariyama, T. , & Yamamoto, K. (1982). A comparison of electrophysiologically determined spectral responses in 35 species of Lepidoptera. Journal of Insect Physiology, 28, 675–682. 10.1016/0022-1910(82)90145-7

[ece310899-bib-0023] Ellingson, J. , Fleishman, L. , & Loew, E. (1995). Visual pigments and spectral sensitivity of the diurnal gecko *Gonatodes albogularis* . Journal of Comparative Physiology A, 177, 559–567. 10.1007/BF00207185 7473305

[ece310899-bib-0024] Endler, J. A. (1984). Natural and sexual selection on color patterns in poeciliid fishes. In T. M. Zarct (Ed.), Evolutionary ecology of neotropical freshwater fishes (pp. 95–111). Springer.

[ece310899-bib-0025] Endler, J. A. (1993). The color of light in forests and its implications. Ecological Monographs, 63, 2–27. 10.2307/2937121

[ece310899-bib-0026] Enright, J. M. , Toomey, M. B. , Sato, S.‐y. , Temple, S. E. , Allen, J. R. , Fujiwara, R. , Kramlinger, V. M. , Nagy, L. D. , Johnson, K. M. , Xiao, Y. , How, M. J. , Johnson, S. L. , Roberts, N. W. , Kefalov, V. J. , Guengerich, F. P. , & Corbo, J. C. (2015). Red‐shifts the spectral sensitivity of photoreceptors by converting vitamin A1 into A2. Current Biology, 25, 3048–3057. 10.1016/j.cub.2015.10.018 26549260 PMC4910640

[ece310899-bib-0027] Escobar‐Camacho, D. , Carleton, K. L. , Narain, D. W. , & Pierotti, M. E. (2020). Visual pigment evolution in Characiformes: The dynamic interplay of teleost whole‐genome duplication, surviving opsins and spectral tuning. Molecular Ecology, 29, 2234–2253. 32421918 10.1111/mec.15474 PMC7451407

[ece310899-bib-0028] Escobar‐Camacho, D. , Pierotti, M. E. , Ferenc, V. , Sharpe, D. M. , Ramos, E. , Martins, C. , & Carleton, K. L. (2019). Variable vision in variable environments: The visual system of an invasive cichlid (*Cichla monoculus*) in Lake Gatun, Panama. Journal of Experimental Biology, 222, jeb188300. 30787138 10.1242/jeb.188300 PMC6451412

[ece310899-bib-0029] Fleishman, L. , Bowman, M. , Saunders, D. , Miller, W. , Rury, M. , & Loew, E. (1997). The visual ecology of Puerto Rican anoline lizards: Habitat light and spectral sensitivity. Journal of Comparative Physiology A, 181, 446–460.

[ece310899-bib-0030] Fox, J. , & Weisberg, S. (2019). An R companion to applied regression (2nd ed.). Sage.

[ece310899-bib-0031] Frank, T. M. , Johnsen, S. , Bracken‐Grissom, H. , Messing, C. G. , & Widder, E. (2016). Vision and bioluminescence in the deep‐sea benthos (p. ME34A‐0789). American Geophysical Union.

[ece310899-bib-0032] Frank, T. M. , Johnsen, S. , & Cronin, T. W. (2012). Light and vision in the deep‐sea benthos: II. Vision in deep‐sea crustaceans. Journal of Experimental Biology, 215, 3344–3353. 22956247 10.1242/jeb.072033

[ece310899-bib-0033] Hart, N. S. (2001). The visual ecology of avian photoreceptors. Progress in Retinal and Eye Research, 20, 675–703. 10.1016/S1350-9462(01)00009-X 11470455

[ece310899-bib-0034] Hart, N. S. , & Hunt, D. M. (2007). Avian visual pigments: Characteristics, spectral tuning, and evolution. The American Naturalist, 169, S7–S26. 10.1086/510141 19426092

[ece310899-bib-0035] Hart, N. S. , Lamb, T. D. , Patel, H. R. , Chuah, A. , Natoli, R. C. , Hudson, N. J. , Cutmore, S. C. , Davies, W. I. , Collin, S. P. , & Hunt, D. M. (2020). Visual opsin diversity in sharks and rays. Molecular Biology and Evolution, 37, 811–827. 31770430 10.1093/molbev/msz269

[ece310899-bib-0036] Hart, N. S. , & Vorobyev, M. (2005). Modelling oil droplet absorption spectra and spectral sensitivities of bird cone photoreceptors. Journal of Comparative Physiology A, 191, 381–392. 10.1007/s00359-004-0595-3 15711964

[ece310899-bib-0037] Hill, G. E. (2006). Female mate choice for ornamental coloration. Bird Coloration, 2, 137–200.

[ece310899-bib-0038] Houde, A. E. (1987). Mate choice based upon naturally occurring color‐pattern variation in a guppy population. Evolution, 41, 1–10. 28563755 10.1111/j.1558-5646.1987.tb05766.x

[ece310899-bib-0039] Jacobs, G. H. (2009). Evolution of colour vision in mammals. Philosophical Transactions of the Royal Society, B: Biological Sciences, 364, 2957–2967. 10.1098/rstb.2009.0039 PMC2781854 19720656

[ece310899-bib-0040] Jokela‐Määttä, M. , Smura, T. , Aaltonen, A. , Ala‐Laurila, P. , & Donner, K. (2007). Visual pigments of Baltic Sea fishes of marine and limnic origin. Visual Neuroscience, 24, 389–398. 17822578 10.1017/S0952523807070459

[ece310899-bib-0041] Jones, R. , Pineda, M.‐C. , Luter, H. M. , Fisher, R. , Francis, D. , Klonowski, W. , & Slivkoff, M. (2021). Underwater light characteristics of turbid coral reefs of the inner central great barrier reef. Frontiers in Marine Science, 8, 1–16. 10.3389/fmars.2021.727206 35685121

[ece310899-bib-0042] Kelber, A. (1999). Ovipositing butterflies use a red receptor to see green. Journal of Experimental Biology, 202, 2619–2630. 10482721 10.1242/jeb.202.19.2619

[ece310899-bib-0043] Kelber, A. , & Roth, L. S. V. (2006). Nocturnal colour vision – Not as rare as we might think. Journal of Experimental Biology, 209, 781–788. 10.1242/jeb.02060 16481567

[ece310899-bib-0044] Kelber, A. , Vorobyev, M. , & Osorio, D. (2003). Animal colour vision–behavioural tests and physiological concepts. Biological Reviews, 78, 81–118. 12620062 10.1017/s1464793102005985

[ece310899-bib-0045] Koskelainen, A. , Ala‐Laurila, P. , Fyhrquist, N. , & Donner, K. (2000). Measurement of thermal contribution to photoreceptor sensitivity. Nature, 403, 220–223. 10646610 10.1038/35003242

[ece310899-bib-0046] Kwiatkowski, M. A. , & Sullivan, B. K. (2002). Geographic variation in sexual selection among populations of an iguanid lizard, *Sauromalus obesus* (= ater). Evolution, 56, 2039–2051. 12449491 10.1111/j.0014-3820.2002.tb00130.x

[ece310899-bib-0047] Liebman, P. A. , & Entine, G. (1968). Visual pigments of frog and tadpole (*Rana pipiens*). Vision Research, 8, 761–767. 10.1016/0042-6989(68)90128-4 5664012

[ece310899-bib-0048] Lind, O. , Henze, M. J. , Kelber, A. , & Osorio, D. (2017). Coevolution of coloration and colour vision? Philosophical Transactions of the Royal Society, B: Biological Sciences, 372, 20160338. 10.1098/rstb.2016.0338 PMC5444059 28533455

[ece310899-bib-0049] Loew, E. R. , & McFarland, W. N. (1990). The underwater visual environment. In R. Douglas & M. Djamgoz (Eds.), The visual system of fish. (pp. 1–43). Springer.

[ece310899-bib-0050] Luo, D.‐G. , Yue, W. W. , Ala‐Laurila, P. , & Yau, K.‐W. (2011). Activation of visual pigments by light and heat. Science, 332, 1307–1312. 21659602 10.1126/science.1200172 PMC4349410

[ece310899-bib-0051] Lythgoe, J. , Muntz, W. , Partridge, J. , Shand, J. , & Williams, D. M. (1994). The ecology of the visual pigments of snappers (Lutjanidae) on the great barrier reef. Journal of Comparative Physiology A, 174, 461–467.

[ece310899-bib-0052] Lythgoe, J. N. (1972). The adaptation of visual pigments to the photic environment. In H. J. A. Dartnall (Ed.), Photochemistry of vision (pp. 566–603). Springer.

[ece310899-bib-0053] Lythgoe, J. N. (1979). The ecology of vision. Clarendon Press.

[ece310899-bib-0054] Lythgoe, J. N. (1988). Light and vision in the aquatic environment. In J. Atema , R. R. Fay , A. N. Popper , W. N. Tavolga (Eds.), Sensory biology of aquatic animals (pp. 57–82). Springer.

[ece310899-bib-0055] Marshall, N. , Jennings, K. , McFarland, W. , Loew, E. , Losey, G. , & Montgomery, W. (2003). Visual biology of Hawaiian coral reef fishes. III. Environmental light and an integrated approach to the ecology of reef fish vision. Copeia, 2003, 467–480.

[ece310899-bib-0056] Marshall, N. J. , Cronin, T. W. , & Frank, T. M. (2003). Visual adaptations in crustaceans: Chromatic, developmental, and temporal aspects. In S. P. Collin & N. J. Marshall (Eds.), Sensory Processing in Aquatic Environments (pp. 343–372). Springer.

[ece310899-bib-0057] Martin, M. , Le Galliard, J.‐F. , Meylan, S. , & Loew, E. R. (2015). The importance of ultraviolet and near‐infrared sensitivity for visual discrimination in two species of lacertid lizards. Journal of Experimental Biology, 218, 458–465. 25524990 10.1242/jeb.115923

[ece310899-bib-0058] Meinertzhagen, I. , Menzel, R. , & Kahle, G. (1983). The identification of spectral receptor types in the retina and lamina of the dragonfly *Sympetrum rubicundulum* . Journal of Comparative Physiology, 151, 295–310.

[ece310899-bib-0059] Mollon, J. D. (1989). “Tho'she kneel'd in that place where they grew…” the uses and origins of primate colour vision. Journal of Experimental Biology, 146, 21–38. 2689563 10.1242/jeb.146.1.21

[ece310899-bib-0060] Murphy, M. J. , & Westerman, E. L. (2022a). Data from: Evolutionary history limits species' ability to match colour sensitivity to available habitat light . Dryad Digital Repository. 10.1098/rspb.2022.0612 PMC9115023 35582803

[ece310899-bib-0061] Murphy, M. J. , & Westerman, E. L. (2022b). Evolutionary history limits species' ability to match colour sensitivity to available habitat light. Proceedings of the Royal Society B: Biological Sciences, 289, 20220612. 10.1098/rspb.2022.0612 PMC9115023 35582803

[ece310899-bib-0062] Nagloo, N. , Collin, S. P. , Hemmi, J. M. , & Hart, N. S. (2016). Spatial resolving power and spectral sensitivity of the saltwater crocodile, *Crocodylus porosus*, and the freshwater crocodile, *Crocodylus johnstoni* . Journal of Experimental Biology, 219, 1394–1404. 27208035 10.1242/jeb.135673

[ece310899-bib-0063] O'Day, W. T. , & Fernandez, H. R. (1974). Aristostomias scintillans (Malacosteidae): A deep‐sea fish with visual pigments apparently adapted to its own bioluminescence. Vision Research, 14, 545–550. 4424870 10.1016/0042-6989(74)90044-3

[ece310899-bib-0064] Ogawa, Y. , Kinoshita, M. , Stavenga, D. G. , & Arikawa, K. (2013). Sex‐specific retinal pigmentation results in sexually dimorphic long‐wavelength‐sensitive photoreceptors in the eastern pale clouded yellow butterfly, *Colias erate* . Journal of Experimental Biology, 216, 1916–1923. 23393285 10.1242/jeb.083485

[ece310899-bib-0065] Osorio, D. , & Bossomaier, T. (1992). Human cone‐pigment spectral sensitivities and the reflectances of natural surfaces. Biological Cybernetics, 67, 217–222. 1498187 10.1007/BF00204394

[ece310899-bib-0066] Osorio, D. , & Vorobyev, M. (2005). Photoreceptor spectral sensitivities in terrestrial animals: Adaptations for luminance and colour vision. Proceedings of the Royal Society B: Biological Sciences, 272, 1745–1752. 10.1098/rspb.2005.3156 PMC1559864 16096084

[ece310899-bib-0067] Osorio, D. , & Vorobyev, M. (2008). A review of the evolution of animal colour vision and visual communication signals. Vision Research, 48, 2042–2051. 18627773 10.1016/j.visres.2008.06.018

[ece310899-bib-0068] Partridge, J. (1989). The visual ecology of avian cone oil droplets. Journal of Comparative Physiology A, 165, 415–426.

[ece310899-bib-0069] Partridge, J. C. , & Douglas, R. H. (1995). Far‐red sensitivity of dragon fish. Nature, 375, 21–22. 10.1038/375021a0

[ece310899-bib-0070] Potier, S. , Mitkus, M. , & Kelber, A. (2020). Visual adaptations of diurnal and nocturnal raptors. Seminars in Cell & Developmental Biology, 106, 116–126. 10.1016/j.semcdb.2020.05.004 32654971

[ece310899-bib-0071] R Core Team . (2022). R: A language and environment for statistical computing. R Foundation for Statistical Computing.

[ece310899-bib-0072] Satoh, A. , Stewart, F. J. , Koshitaka, H. , Akashi, H. D. , Pirih, P. , Sato, Y. , & Arikawa, K. (2017). Red‐shift of spectral sensitivity due to screening pigment migration in the eyes of a moth, *Adoxophyes orana* . Zoological Letters, 3, 14. 10.1186/s40851-017-0075-6 28861276 PMC5575869

[ece310899-bib-0073] Schweikert, L. E. , Caves, E. M. , Solie, S. E. , Sutton, T. T. , & Johnsen, S. (2019). Variation in rod spectral sensitivity of fishes is best predicted by habitat and depth. Journal of Fish Biology, 95, 179–185. 30393870 10.1111/jfb.13859

[ece310899-bib-0074] Sharkey, C. R. , Powell, G. S. , & Bybee, S. M. (2021). Opsin evolution in flower‐visiting beetles. Frontiers in Ecology and Evolution, 9, 1–9. 10.3389/fevo.2021.676369

[ece310899-bib-0075] Sigmund, W. R. (1983). Female preference for *Anolis carolinensis* males as a function of dewlap color and background coloration. Journal of Herpetology, 17, 137–143.

[ece310899-bib-0076] Stieb, S. M. , Cortesi, F. , Jardim De Queiroz, L. , Carleton, K. L. , Seehausen, O. , & Marshall, N. J. (2023). Long‐wavelength‐sensitive (LWS) opsin gene expression, foraging and visual communication in coral reef fishes. Molecular Ecology, 32, 1656–1672. 36560895 10.1111/mec.16831 PMC10065935

[ece310899-bib-0077] Sumner, P. , & Mollon, J. D. (2000). Catarrhine photopigments are optimized for detecting targets against a foliage background. Journal of Experimental Biology, 203, 1963–1986. 10851115 10.1242/jeb.203.13.1963

[ece310899-bib-0078] Sundarabalan, B. , Shanmugam, P. , & Ahn, Y.‐H. (2016). Modeling the underwater light field fluctuations in coastal oceanic waters: Validation with experimental data. Ocean Science Journal, 51, 67–86. 10.1007/s12601-016-0007-y

[ece310899-bib-0079] Surridge, A. K. , Osorio, D. , & Mundy, N. I. (2003). Evolution and selection of trichromatic vision in primates. Trends in Ecology & Evolution, 18, 198–205.

[ece310899-bib-0080] Turner, J. R. , White, E. M. , Collins, M. A. , Partridge, J. C. , & Douglas, R. H. (2009). Vision in lanternfish (Myctophidae): Adaptations for viewing bioluminescence in the deep‐sea. Deep Sea Research Part I: Oceanographic Research Papers, 56, 1003–1017. 10.1016/j.dsr.2009.01.007

[ece310899-bib-0081] van der Kooi, C. J. , Stavenga, D. G. , Arikawa, K. , Belušič, G. , & Kelber, A. (2021). Evolution of insect color vision: From spectral sensitivity to visual ecology. Annual Review of Entomology, 66, 435–461. 10.1146/annurev-ento-061720-071644 32966103

[ece310899-bib-0082] Vranken, N. , Van Steenberge, M. , Kayenbergh, A. , & Snoeks, J. (2020). The lobed‐lipped species of *Haplochromis* (Teleostei, Cichlidae) from Lake Edward, two instead of one. Journal of Great Lakes Research, 46, 1079–1089.

[ece310899-bib-0083] Wakakuwa, M. , Stavenga, D. G. , Kurasawa, M. , & Arikawa, K. (2004). A unique visual pigment expressed in green, red and deep‐red receptors in the eye of the small white butterfly, Pieris Rapae crucivora. Journal of Experimental Biology, 207, 2803–2810. 10.1242/jeb.01078 15235009

[ece310899-bib-0084] Wang, L.‐Y. , Stuart‐Fox, D. , Walker, G. , Roberts, N. W. , & Franklin, A. M. (2022). Insect visual sensitivity to long wavelengths enhances colour contrast of insects against vegetation. Scientific Reports, 12, 982. 10.1038/s41598-021-04702-w 35046431 PMC8770459

[ece310899-bib-0085] Warrant, E. J. , & Johnsen, S. (2013). Vision and the light environment. Current Biology, 23, R990–R994. 24262832 10.1016/j.cub.2013.10.019

[ece310899-bib-0086] Warrant, E. J. , & Locket, N. A. (2004). Vision in the deep sea. Biological Reviews, 79, 671–712. 10.1017/S1464793103006420 15366767

