# Supplementary material for: Red vision in animals is broadly associated with lighting environment but not types of visual task
Source: Ecol Evol. 2024 Jan 31;14(2):e10899. doi: 10.1002/ece3.10899 (PMC10828735; doi:10.1002/ece3.10899)
Supplement: Supplementary file 1 — Table S1: [file ECE3-14-e10899-s001.docx]

**Appendix**

**Table S1: All species or populations identified to possess a long wavelength sensitive (LWS) photoreceptor (λ_max_ ≥ 550 nm)**. Habitat indicates broad-scale habitat (aquatic, semi-aquatic or terrestrial), λ_max(LWS)_ indicates peak sensitivity of the LWS photoreceptor, and all photoreceptors identified for each record are detailed in the photoreceptors column. For full dataset including environmental and ecological variables, see data on Dryad.

| **Class** | **Family** | **Species** | **Habitat** | λ_max(LWS)_ | **Photoreceptors** |
| --- | --- | --- | --- | --- | --- |
| Actinopterygii | Acipenseridae | *Acipenser baerii* | Aquatic | 613 | 465, 549, 613 |
| Actinopterygii | Bryconidae | *Brycon chagrensis* | Aquatic | 612 | 457, 550, 588, 612 |
| Actinopterygii | Chanidae | *Chanos chanos* | Aquatic | 578 | 423, 468, 506, 578 |
| Actinopterygii | Chanidae | *Chanos chanos* | Aquatic | 580 | 441, 502, 580 |
| Actinopterygii | Characidae | *Bryconamericus gonzalezi* | Aquatic | 576 | 456, 536, 576 |
| Actinopterygii | Cichlidae | *Astatotilapia burtoni* | Aquatic | 562 | 455, 523, 562 |
| Actinopterygii | Cichlidae | *Dimidiochromis compressiceps* | Aquatic | 567 | 447, 533, 567 |
| Actinopterygii | Cichlidae | *Haplochromis argens* | Aquatic | 567 | 456, 533, 567 |
| Actinopterygii | Cichlidae | *Haplochromis ishmaeli* | Aquatic | 569 | 456, 535, 569 |
| Actinopterygii | Cichlidae | *Haplochromis pyrrhocephalus* | Aquatic | 595 | 462, 539, 595 |
| Actinopterygii | Cichlidae | *Haplochromis piceatus* | Aquatic | 565 | 465, 522, 565 |
| Actinopterygii | Cichlidae | *Lethrinops parvidens* | Aquatic | 563 | 426, 514, 563 |
| Actinopterygii | Cichlidae | *Oreochromis niloticus* | Aquatic | 595 | 359, 427, 456, 483, 529, 595 |
| Actinopterygii | Cichlidae | *Protomelas taeniolatus* | Aquatic | 566 | 453, 527, 566 |
| Actinopterygii | Cichlidae | *Pundamilia azurea* | Aquatic | 564 | 456, 526, 564 |
| Actinopterygii | Cichlidae | *Pundamilia nyererei (Makobe population)* | Aquatic | 565 | 528, 565 |
| Actinopterygii | Cichlidae | *Pundamilia nyererei (Python population)* | Aquatic | 568 | 452, 535, 568 |
| Actinopterygii | Cichlidae | *Pundamilia pundamilia (Makobe population)* | Aquatic | 564 | 456, 527, 564 |
| Actinopterygii | Cichlidae | *Pundamilia pundamilia (Python population)* | Aquatic | 563 | 455, 529, 563 |
| Actinopterygii | Cichlidae | *Tramitichromis intermedius* | Aquatic | 569 | 455, 532, 569 |
| Actinopterygii | Clupeidae | *Clupea pallasii* | Aquatic | 584 | 480, 500, 521, 584 |
| Actinopterygii | Cottidae | *Hemilepidotus hemilepidotus* | Aquatic | 568 | 408, 482, 498, 568 |
| Actinopterygii | Cottidae | *Scorpaenichthys marmoratus* | Aquatic | 569 | 364, 479, 569 |
| Actinopterygii | Cryptacanthodidae | *Cryptacanthodes aleutensis* | Aquatic | 574 | 355, 409, 513, 574 |
| Actinopterygii | Curimatidae | *Cyphocharax magdalenae* | Aquatic | 587 | 453, 543, 587 |
| Actinopterygii | Cyprinidae | *Carrassius auratus* | Aquatic | 614 | 358, 452, 532, 614 |
| Actinopterygii | Cyprinidae | *Danio rerio* | Aquatic | 570 | 360, 415, 480, 570 |
| Actinopterygii | Erythrinidae | *Hoplias microlepis* | Aquatic | 614 | 548, 573, 595 |
| Actinopterygii | Fundulidae | *Lucania goodei* | Aquatic | 573 | 359, 405, 455, 539, 573 |
| Actinopterygii | Gasterosteidae | *Gasterosteus aculeatus* | Aquatic | 605 | 360, 445, 530, 605 |
| Actinopterygii | Hemitripteridae | *Blepsias cirrhosus* | Aquatic | 569 | 420, 435, 521, 569 |
| Actinopterygii | Hexagrammidae | *Hexagrammos octogrammus* | Aquatic | 570 | 530, 570 |
| Actinopterygii | Hexagrammidae | *Hexagrammos stelleri* | Aquatic | 558 | 364, 519, 556 |
| Actinopterygii | Hexagrammidae | *Ophiodon elongatus* | Aquatic | 562 | 359, 403, 513, 562 |
| Actinopterygii | Labridae | *Thalassoma duperrey* | Aquatic | 570 | 460, 548, 570 |
| Actinopterygii | Lutjanidae | *Lutjanus argentimaculatus* | Aquatic | 575 | 536, 575 |
| Actinopterygii | Lutjanidae | *Lutjanus fulviflamma* | Aquatic | 568 | 505, 534, 568 |
| Actinopterygii | Lutjanidae | *Lutjanus johnii* | Aquatic | 572 | 498, 534, 572 |
| Actinopterygii | Lutjanidae | *Lutjanus johnii* | Aquatic | 567 | 458, 543, 567 |
| Actinopterygii | Lutjanidae | *Lutjanus russelli* | Aquatic | 567 | 534, 567 |
| Actinopterygii | Poeciliidae | *Poecilia formosa* | Aquatic | 560 | 352, 404, 463, 560 |
| Actinopterygii | Poeciliidae | *Poecilia latipinna* | Aquatic | 577 | 403, 463, 551, 577 |
| Actinopterygii | Poeciliidae | *Poecilia mexicana* | Aquatic | 563 | 350, 403, 461, 537, 563 |
| Actinopterygii | Poeciliidae | *Poecilia mexicana (cave population)* | Aquatic | 572 | 374, 404, 456, 532, 572 |
| Actinopterygii | Poeciliidae | *Poecilia reticulata (Cumaná)* | Aquatic | 560 | 359, 406, 465, 525, 541, 560 |
| Actinopterygii | Poeciliidae | *Poecilia reticulata (Trinidad)* | Aquatic | 572 | 389, 408, 464, 533, 548, 572 |
| Actinopterygii | Poeciliidae | *Xiphophorus helleri* | Aquatic | 568 | 365, 405, 459, 534, 568 |
| Actinopterygii | Salmonidae | *Oncorhynchus mykiss* | Aquatic | 576 | 365, 434, 531, 576 |
| Actinopterygii | Salmonidae | *Salmo trutta* | Aquatic | 600 | 355, 440, 535, 600 |
| Actinopterygii | Stomiidae | *Aristostomias scintillans* | Aquatic | 551 | 526, 551 |
| Actinopterygii | Stomiidae | *Aristostomias grimaldii* | Aquatic | 552 | 517, 552 |
| Actinopterygii | Stomiidae | *Aristostomias tittmanni* | Aquatic | 581 | 518, 550, 581 |
| Actinopterygii | Stomiidae | *Malacosteus danae* | Aquatic | 556 | 514, 556 |
| Actinopterygii | Stomiidae | *Pachystomias microdon* | Aquatic | 595 | 520, 563, 595 |
| Amphibia | Ambystomatidae | *Ambystoma tigrinum* | Aquatic | 610 | 400, 444, 610 |
| Amphibia | Ranidae | *Rana pipiens* | Aquatic | 620 | 527, 620 |
| Amphibia | Ranidae | *Rana pipiens* | Semi-aquatic | 575 | 502, 575 |
| Amphibia | Ranidae | *Rana temporaria* | Semi-aquatic | 562 | 431, 502, 562 |
| Amphibia | Salamandridae | *Cynops orientalis* | Semi-aquatic | 601 | 350, 474, 596 |
| Amphibia | Salamandridae | *Lissotriton (Triturus) vulgaris* | Semi-aquatic | 609 | 340, 470, 609 |
| Amphibia | Salamandridae | *Pleurodeles waltl* | Semi-aquatic | 611 | 361, 489, 611 |
| Arachnida | Salticidae | *Habronattus pyrrithrix* | Terrestrial | 626 | 377, 530, 626 |
| Arachnida | Salticidae | *Menemerus fulvus* | Terrestrial | 580 | 360, 490, 520, 580 |
| Branchiopoda | Daphniidae | *Daphnia magna* | Aquatic | 608 | 348, 434, 525, 608 |
| Chondrichthyes | Carcharhinidae | *Carcharhinus leucas* | Aquatic | 554 | 554 |
| Chondrichthyes | Dasyatidae | *Dasyatis fluviorum* | Aquatic | 566 | 475, 566 |
| Chondrichthyes | Dasyatidae | *Neotrygon kuhlii* | Aquatic | 552 | 476, 552 |
| Chondrichthyes | Dasyatidae | *Pateobatis fai* | Aquatic | 557 | 475, 557 |
| Chondrichthyes | Dasyatidae | *Taeniura lymma* | Aquatic | 557 | 479, 557 |
| Chondrichthyes | Gymnuridae | *Gymnura australis* | Aquatic | 556 | 467, 556 |
| Chondrichthyes | Myliobatidae | *Aetobatus narinari* | Aquatic | 552 | 450, 552 |
| Chondrichthyes | Orectolobidae | *Orectolobus maculatus* | Aquatic | 553 | 553 |
| Chondrichthyes | Orectolobidae | *Orectolobus ornatus* | Aquatic | 561 | 561 |
| Chondrichthyes | Rhinobatidae | *Aptychotrema rostrata* | Aquatic | 553 | 459, 553 |
| Chondrichthyes | Rhinobatidae | *Aptychotrema vincentiana* | Aquatic | 557 | 460, 557 |
| Chondrichthyes | Rhinobatidae | *Glaucostegus typus* | Aquatic | 561 | 477, 561 |
| Chondrichthyes | Rhinobatidae | *Trygonorrhina fasciata* | Aquatic | 568 | 470, 568 |
| Chondrichthyes | Rhinopteridae | *Rhinoptera bonasus* | Aquatic | 551 | 470, 551 |
| Chondrichthyes | Rhynchobatidae | *Rhynchobatus australiae* | Aquatic | 555 | 475, 555 |
| Chondrichthyes | Urolophidae | *Trygonoptera testacea* | Aquatic | 557 | 481, 557 |
| Chondrichthyes | Urotrygonidae | *Urobatis jamaicensis* | Aquatic | 562 | 475, 562 |
| Hyperoartia | Petromyzontidae | *Lampetra lampetra fluviatilis* | Aquatic | 555 | 555 |
| Insecta | Aeschnidae | *Aeshna cyanea* | Aquatic | 615 | 356, 412, 519, 615 |
| Insecta | Aeschnidae | *Aeshna mixta* | Aquatic | 615 | 356, 412, 519, 615 |
| Insecta | Andrenidae | *Callonychium petuniae* | Terrestrial | 593 | 356, 445, 531, 593 |
| Insecta | Apidae | *Apis mellifera* | Terrestrial | 556 | 344, 436, 556 |
| Insecta | Buprestidae | *Coroebus undatus* | Terrestrial | 600 | 335, 350, 430, 540, 600 |
| Insecta | Buprestidae | *Agrilus planipennis* | Terrestrial | 550 | 340, 425, 460, 550 |
| Insecta | Calopterygidae | *Calypteryx splendens and virgo* | Terrestrial | 640 | 366, 480, 552, 640 |
| Insecta | Carabidae | *Carabus auratus* | Terrestrial | 620 | 348, 430, 500, 620 |
| Insecta | Carabidae | *Carabus granulatus* | Terrestrial | 620 | 348, 430, 500, 620 |
| Insecta | Carabidae | *Carabus nemoralis* | Terrestrial | 620 | 348, 430, 500, 620 |
| Insecta | Castnidaee | *Paysandisia archon* | Terrestrial | 580 | 360, 465, 550, 580 |
| Insecta | Coenagrionidae | *Ischnura elegans* | Terrestrial | 600 | 370, 440, 540, 600 |
| Insecta | Corduliidae | *Hemicordulia tau* | Terrestrial | 630 | 330, 410, 460, 525, 630 |
| Insecta | Culicidae | *Aedes aegypti* | Terrestrial | 621 | 334, 523, 621 |
| Insecta | Diopsidae | *Teleopsis dalmanni* | Terrestrial | 660 | 360, 450, 490, 660 |
| Insecta | Formicidae | *Myrmecia vindex* | Terrestrial | 550 | 370, 450, 550 |
| Insecta | Formicidae | *Solenopsis saevissima* | Terrestrial | 620 | 360, 505, 620 |
| Insecta | Glaphyridae | *Pygopleurus israelitus* | Terrestrial | 631 | 360, 517, 631 |
| Insecta | Hepialidae | *Endoclita excrescens* | Terrestrial | 580 | 400, 450, 530, 580 |
| Insecta | Lampyridae | *Photuris lucicrescens* | Terrestrial | 550 | 350, 440, 550 |
| Insecta | Libellulidae | *Sympetrum rubicundulum* | Terrestrial | 620 | 340, 410, 490, 540, 620 |
| Insecta | Lycaenidae | *Lycaena phlaeas* | Terrestrial | 600 | 400, 540, 600 |
| Insecta | Lycaenidae | *Pseudozizeeria maha* | Terrestrial | 580 | 400, 520, 580 |
| Insecta | Megachilidae | *Osmia rufa* | Terrestrial | 560 | 344, 432, 560 |
| Insecta | Noctuidae | *Mamestra brassicae* | Terrestrial | 580 | 360, 460, 540, 580 |
| Insecta | Nymphalidae | *Argyronome ruslana* | Terrestrial | 620 | 380, 440, 560, 620 |
| Insecta | Nymphalidae | *Fabriciana adippe* | Terrestrial | 580 | 380, 460, 520, 580 |
| Insecta | Nymphalidae | *Heliconius erato* | Terrestrial | 600 | 360, 390, 470, 560, 600 |
| Insecta | Nymphalidae | *Heliconius erato* | Terrestrial | 600 | 390, 470, 560, 600 |
| Insecta | Nymphalidae | *Polygonia c-aureum* | Terrestrial | 580 | 340, 440, 460, 520, 560, 580 |
| Insecta | Papillionidae | *Atrophaneura alcinous* | Terrestrial | 600 | 420, 460, 520, 600 |
| Insecta | Papillionidae | *Graphium sarpedon* | Terrestrial | 640 | 360, 400, 460, 480, 500, 540, 560, 580, 600, 640 |
| Insecta | Papillionidae | *Papilio aegeus* | Terrestrial | 610 | 360, 390, 450, 540, 610 |
| Insecta | Papillionidae | *Papilio bianor* | Terrestrial | 580 | 420, 460, 520, 580 |
| Insecta | Papillionidae | *Papilio maackii* | Terrestrial | 580 | 380, 460, 520, 580 |
| Insecta | Papillionidae | *Papilio machaon* | Terrestrial | 580 | 380, 460, 520, 580 |
| Insecta | Papillionidae | *Papilio xuthus* | Terrestrial | 600 | 360, 400, 460, 520, 600 |
| Insecta | Papillionidae | *Troides aeacus formosanum* | Terrestrial | 630 | 360, 390, 440, 510, 540, 550, 580, 610, 630 |
| Insecta | Pieridae | *Colias erate* | Terrestrial | 660 | 360, 440, 480, 580, 660 |
| Insecta | Pieridae | *Colias erate* | Terrestrial | 660 | 360, 430, 460, 580, 620, 640, 660 |
| Insecta | Pieridae | *Gonepteryx aspasia* | Terrestrial | 620 | 380, 460, 560, 620 |
| Insecta | Pieridae | *Pieris brassicae* | Terrestrial | 620 | 360, 455, 560, 620 |
| Insecta | Pieridae | *Pieris melete* | Terrestrial | 600 | 400, 480, 540, 600 |
| Insecta | Pieridae | *Pieris rapae crucivora* | Terrestrial | 640 | 360, 440, 460, 560, 620, 640 |
| Insecta | Pieridae | *Pieris rapae crucivora* | Terrestrial | 640 | 360, 420, 440, 560, 620, 640 |
| Insecta | Rhagophthalmidae | *Rhagophthalmus ohbai* | Terrestrial | 600 | 360, 550, 600 |
| Insecta | Riodinidae | *Apodemia mormo* | Terrestrial | 600 | 505, 600 |
| Insecta | Saturniidae | *Actias artemis* | Terrestrial | 580 | 380, 460, 540, 580 |
| Insecta | Sphingidae | *Marumba sperchius* | Terrestrial | 600 | 460, 540, 600 |
| Insecta | Sphingidae | *Ampelophaga rubiginosa* | Terrestrial | 580 | 460, 540, 580 |
| Insecta | Sphingidae | *Callambulyx tatarinovii* | Terrestrial | 580 | 380, 460, 540, 580 |
| Insecta | Tenthredinidae | *Tenthredo campestris* | Terrestrial | 602 | 337, 458, 537, 602 |
| Insecta | Tenthredinidae | *Tenthredo scrophulariae* | Terrestrial | 592 | 332, 592 |
| Insecta | Tortricidae | *Cydia pomonella* | Terrestrial | 580 | 365, 550, 580 |
| Insecta | Xiphydriidae | *Xiphydria camelus* | Terrestrial | 604 | 556, 604 |
| Malacostraca | Cambaridae | *Faxonius virilis* | Aquatic | 565 | 425, 565 |
| Malacostraca | Cambaridae | *Procambarus clarkii* | Aquatic | 600 | 460, 600 |
| Malacostraca | Mysidae | *Mysis relicta* | Aquatic | 556 | 556 |
| Malacostraca | Mysidae | *Mysis salemaai* | Aquatic | 557 | 557 |
| Malacostraca | Mysidae | *Mysis segerstralei* | Aquatic | 562 | 562 |
| Malacostraca | Palaemonidae | *Palaemonetes paludosus* | Aquatic | 555 | 380, 555 |
| Malacostraca | Gonodactylidae | *Neogonodactylus oerstedii* | Aquatic | 551 | 401, 422, 424, 459, 488, 489, 503, 522, 525, 528, 551 |
| Reptilia | Agamidae | *Ctenophorus decresii.* | Terrestrial | 569 | 436, 495, 569 |
| Reptilia | Agamidae | *Ctenophorus ornatus* | Terrestrial | 571 | 440, 493, 571 |
| Reptilia | Alligatoridae | *Alligator mississippiensis* | Semi-aquatic | 566 | 444, 535, 566 |
| Reptilia | Boidae | *Boa constrictor* | Terrestrial | 549 | 357, 495, 549 |
| Reptilia | Chamaeleonidae | *Chamaeleo dilepis* | Terrestrial | 615 | 385, 440, 497, 605 |
| Reptilia | Chamaeleonidae | *Furcifer pardalis* | Terrestrial | 610 | 375, 444, 490, 605 |
| Reptilia | Colubridae | *Thamnophis sirtalis sirtalis* | Terrestrial | 554 | 358, 481, 554 |
| Reptilia | Cordylidae | *Platysaurus broadleyi* | Terrestrial | 570 | 364, 451, 492, 570 |
| Reptilia | Crocodylidae | *Crocodylus johnstoni* | Semi-aquatic | 554 | 426, 510, 554 |
| Reptilia | Crotaphytidae | *Crotaphytus dickersonae* | Terrestrial | 558 | 359, 459, 481, 558 |
| Reptilia | Dactyloidae | *Anolis bahorucoensis* | Terrestrial | 569 | 365, 450, 500, 569 |
| Reptilia | Dactyloidae | *Anolis carolinensis* | Terrestrial | 625 | 365, 462, 503, 625 |
| Reptilia | Dactyloidae | *Anolis conspersus* | Terrestrial | 562 | 365, 460, 500, 562 |
| Reptilia | Dactyloidae | *Anolis cristatellus* | Terrestrial | 562 | 365, 458, 492, 562 |
| Reptilia | Dactyloidae | *Anolis equetris* | Terrestrial | 565 | 460, 492, 565 |
| Reptilia | Dactyloidae | *Anolis evermanni* | Terrestrial | 565 | 364, 460, 490, 565 |
| Reptilia | Dactyloidae | *Anolis extremus* | Terrestrial | 566 | 365, 451, 487, 566 |
| Reptilia | Dactyloidae | *Anolis garmani* | Terrestrial | 565 | 467, 496, 565 |
| Reptilia | Dactyloidae | *Anolis grahami* | Terrestrial | 565 | 367, 460, 495, 565 |
| Reptilia | Dactyloidae | *Anolis gundlachi* | Terrestrial | 564 | 365, 450, 490, 564 |
| Reptilia | Dactyloidae | *Anolis krugi* | Terrestrial | 562 | 365, 448, 490, 562 |
| Reptilia | Dactyloidae | *Anolis lineatopus* | Terrestrial | 560 | 366, 449, 498, 560 |
| Reptilia | Dactyloidae | *Anolis opalinus* | Terrestrial | 566 | 450, 496, 566 |
| Reptilia | Dactyloidae | *Anolis pulchellus* | Terrestrial | 565 | 367, 446, 495, 565 |
| Reptilia | Dactyloidae | *Anolis sagrei* | Terrestrial | 567 | 365, 460, 495, 567 |
| Reptilia | Dactyloidae | *Anolis stratulus* | Terrestrial | 564 | 366, 454, 494, 564 |
| Reptilia | Dactyloidae | *Anolis valencienni* | Terrestrial | 560 | 456, 500, 560 |
| Reptilia | Emydidae | *Trachemys scripta elegan* | Semi-aquatic | 617 | 372, 458, 515, 617 |
| Reptilia | Polychrotidae | *Polychrus marmoratus* | Terrestrial | 568 | 453, 490, 568 |
| Reptilia | Pythonidae | *Python regius* | Terrestrial | 551 | 360, 494, 551 |
